# Sexually Dimorphic Glutamatergic Control of R-Spondin 1 and R-Spondin 3 Neurons in Hypothalamic Reproductive and Metabolic Nuclei

**DOI:** 10.3390/ijms27209026

**Published:** 2026-10-12

**Authors:** Gonca Topal, Duygu Gok Yurtseven, Nursel Hasanoglu Akbulut, Zulfiye Gul, Duygu Udum, Murat Yalcin, Ozhan Eyigor

**Affiliations:** 1 Department of Histology and Embryology, Faculty of Medicine, Bursa Uludag University, Bursa 16059, Türkiyeoeyigor@uludag.edu.tr (O.E.); 2 Department of Histology and Embryology, Faculty of Medicine, Canakkale Onsekiz Mart University, Çanakkale 17020, Türkiye; nurselhasanoglu@comu.edu.tr; 3 Department of Medical Pharmacology, Faculty of Medicine, Dokuz Eylul University, İzmir 35340, Türkiye; zulfiye.gul@deu.edu.tr; 4 Department of Biochemistry, Faculty of Veterinary Medicine, Bursa Uludag University, Bursa 16059, Türkiye; duygudum@uludag.edu.tr; 5 Department of Veterinary Physiology, Faculty of Veterinary Medicine, Bursa Uludag University, Bursa 16059, Türkiye; muraty@uludag.edu.tr

**Keywords:** R-Spondin 1, R-spondin 3, WNT/β-catenin, LGR4, glutamate receptors, hypothalamus, paraventricular nucleus, sexual dimorphism, c-Fos, reproduction

## Abstract

R-spondin 1 (RSPO1) and R-spondin 3 (RSPO3) contribute to reproductive development and feeding regulation, but their hypothalamic glutamatergic regulation remains unclear. We hypothesized that NMDA and non-NMDA receptor mechanisms contribute to hypothalamic RSPO neuronal activation. Given steroid-sensitive hypothalamic glutamatergic signaling, we compared activation and antagonist-mediated attenuation between sexes. Adult male and metestrous female Sprague–Dawley rats received kainic acid or AMPA with or without CNQX pretreatment, or NMDA with or without MK-801 pretreatment. RSPO/c-Fos labeling assessed activation in the supraoptic (SON), paraventricular (PVN), and arcuate (ARC) nuclei; ELISA assessed accompanying changes in serum RSPO concentrations. All agonists activated both populations in all nuclei. Only kainate-induced PVN RSPO3 activation differed between sexes (females, 89.3%; males, 72.8%; unadjusted *p* = 0.039), an isolated finding among 18 agonist comparisons. Antagonists attenuated all responses, although activation remained above control in 17 of 36 conditions. Females showed greater attenuation in selected kainate and NMDA conditions in the SON and PVN. Serum changes did not parallel neuronal activation. These findings support NMDA and non-NMDA receptor involvement and sex-dependent attenuation of RSPO neuronal activation. Given the central feeding effects of RSPOs, their sex-dependent glutamatergic regulation within neuroendocrine nuclei may contribute to sex differences in metabolic control.

## 1. Introduction

The R-spondin (RSPO) family comprises four secreted glycoproteins, RSPO1–RSPO4, that amplify WNT/β-catenin signaling [1,2,3]. They act principally through leucine-rich repeat-containing G-protein-coupled receptors LGR4–LGR6 and the transmembrane E3 ubiquitin ligases ZNRF3 and RNF43 [4,5,6,7,8]. By inhibiting ZNRF3/RNF43-mediated turnover of WNT receptors, RSPOs increase cellular responsiveness to WNT ligands.

RSPO signaling has established functions in reproductive development. Human RSPO1 loss-of-function mutations cause 46,XX testicular disorder of sex development [9], and *Rspo1* deletion disrupts ovarian differentiation and follicle formation in mice [10,11]. RSPO3 is required for placental vascular development [12,13], while LGR4 deficiency causes abnormalities of reproductive tract development and gonadal function [14,15,16].

Evidence also supports a central role in metabolic regulation. LGR4 is broadly expressed in the central nervous system [17]. In male rats, hypothalamic *Rspo1* and *Rspo3* expression responds to fasting and insulin, and intracerebroventricular administration of either peptide suppresses food intake [18]. Conditional *Lgr4* deletion in the arcuate nucleus (ARC) of mice disrupts RSPO1-induced feeding suppression and reduces pro-opiomelanocortin (*Pomc*) expression [19]. These findings implicate hypothalamic RSPO signaling in feeding regulation and raise the question of how neuronal inputs control the activation of RSPO-producing populations.

Our previous immunohistochemical study identified RSPO1- and RSPO3-expressing neurons in the supraoptic (SON), paraventricular (PVN), and ARC nuclei of adult male rats [20]. These regions contain populations with complementary metabolic and neuroendocrine functions. The ARC includes POMC and neuropeptide Y/agouti-related protein neurons involved in energy balance, as well as kisspeptin/neurokinin B/dynorphin neurons involved in gonadotropin-releasing hormone pulse generation [21,22,23]. The SON and PVN contain oxytocin and vasopressin neurosecretory systems involved in lactation and homeostatic regulation [24,25,26]. The PVN also contains melanocortin-4 receptor (MC4R)-expressing glutamatergic neurons that regulate feeding [27]. This anatomical distribution provides a rationale for examining RSPO neuronal regulation in regions involved in metabolic regulation and reproductive neuroendocrine function.

Glutamate is a major excitatory transmitter in hypothalamic neuroendocrine circuits [28,29]. Its relevance extends to these specific networks: local ionotropic glutamate receptors regulate oxytocin and vasopressin secretion through region-dependent mechanisms in the SON and PVN [30], and POMC-derived α-melanocyte-stimulating hormone potentiates glutamatergic ARC input to PVN MC4R neurons [31]. However, whether NMDA and non-NMDA receptor mechanisms contribute to the activation of RSPO populations in the SON, PVN, and ARC has not been defined. These receptor classes differ in their pharmacological and biophysical properties [32,33,34,35,36]. CNQX antagonizes both AMPA and kainate receptors, whereas MK-801 blocks activated NMDA receptor channels [37,38]. Combining the corresponding agonists and antagonists therefore allows assessment of non-NMDA and NMDA receptor contributions to RSPO neuronal activation and the extent to which these responses can be attenuated.

There is also a biological rationale for comparing males and females. Estradiol can enhance glutamate release and organize synaptic connections in the developing hypothalamus [39], while ovarian steroids regulate glutamate receptor expression in the anteroventral periventricular nucleus [40]. These observations suggest that sex may influence the pharmacological regulation of hypothalamic RSPO populations. Comparing both agonist-induced activation and antagonist-mediated attenuation addresses whether sex differences occur in activation, its inhibition, or both.

We tested the hypothesis that NMDA and non-NMDA glutamate receptor mechanisms contribute to RSPO1 and RSPO3 neuronal activation in the SON, PVN, and ARC. We also examined whether activation and its attenuation differ by sex and hypothalamic region. Adult male and metestrous female rats received systemic kainic acid or AMPA with or without CNQX pretreatment, or NMDA with or without MK-801 pretreatment. Neuronal activation was assessed by dual RSPO/c-Fos labeling [41,42]. Serum RSPO1 and RSPO3 concentrations were also measured by ELISA to characterize the circulating response to systemic glutamatergic treatment and assess whether it paralleled hypothalamic RSPO neuronal activation.

## 2. Results

### 2.1. Identification and Distribution of RSPO-Immunoreactive Neurons

**Figure 1:**
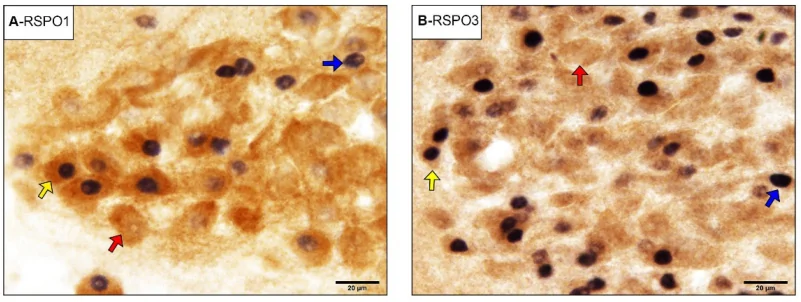
Dual immunoperoxidase labeling of RSPO1 and RSPO3 with c-Fos in the rat hypothalamus. Representative images show (**A**) RSPO1/c-Fos and (**B**) RSPO3/c-Fos labeling. RSPO immunoreactivity is visualized as a brown cytoplasmic DAB reaction product, whereas c-Fos immunoreactivity appears as a blue-black nuclear reaction product following nickel-enhanced DAB staining. Yellow arrows indicate double-labeled neurons; red arrows indicate RSPO-immunoreactive neurons without detectable nuclear c-Fos labeling; blue arrows indicate c-Fos-positive nuclei without detectable surrounding RSPO immunoreactivity. Double-labeled neurons were counted as activated RSPO neurons. Scale bars: 20 µm.

RSPO1 and RSPO3 immunoreactivity appeared as brown cytoplasmic labeling, whereas c-Fos-positive nuclei were blue-black. Neurons containing both signals were scored as activated (Figure 1). RSPO-negative profiles with c-Fos-positive nuclei were also present. RSPO-immunoreactive somata were identified in the SON, PVN, and ARC of both sexes, with the male distribution consistent with our previous mapping [20]. No countable RSPO-positive somata were identified in the ventromedial hypothalamic nucleus (VMH) in the sections examined, and this nucleus was excluded from analysis. No discernible cellular labeling was observed when the primary or secondary antibody was omitted.

### 2.2. Glutamate Receptor Agonists Activate Hypothalamic RSPO Populations

**Figure 2:**
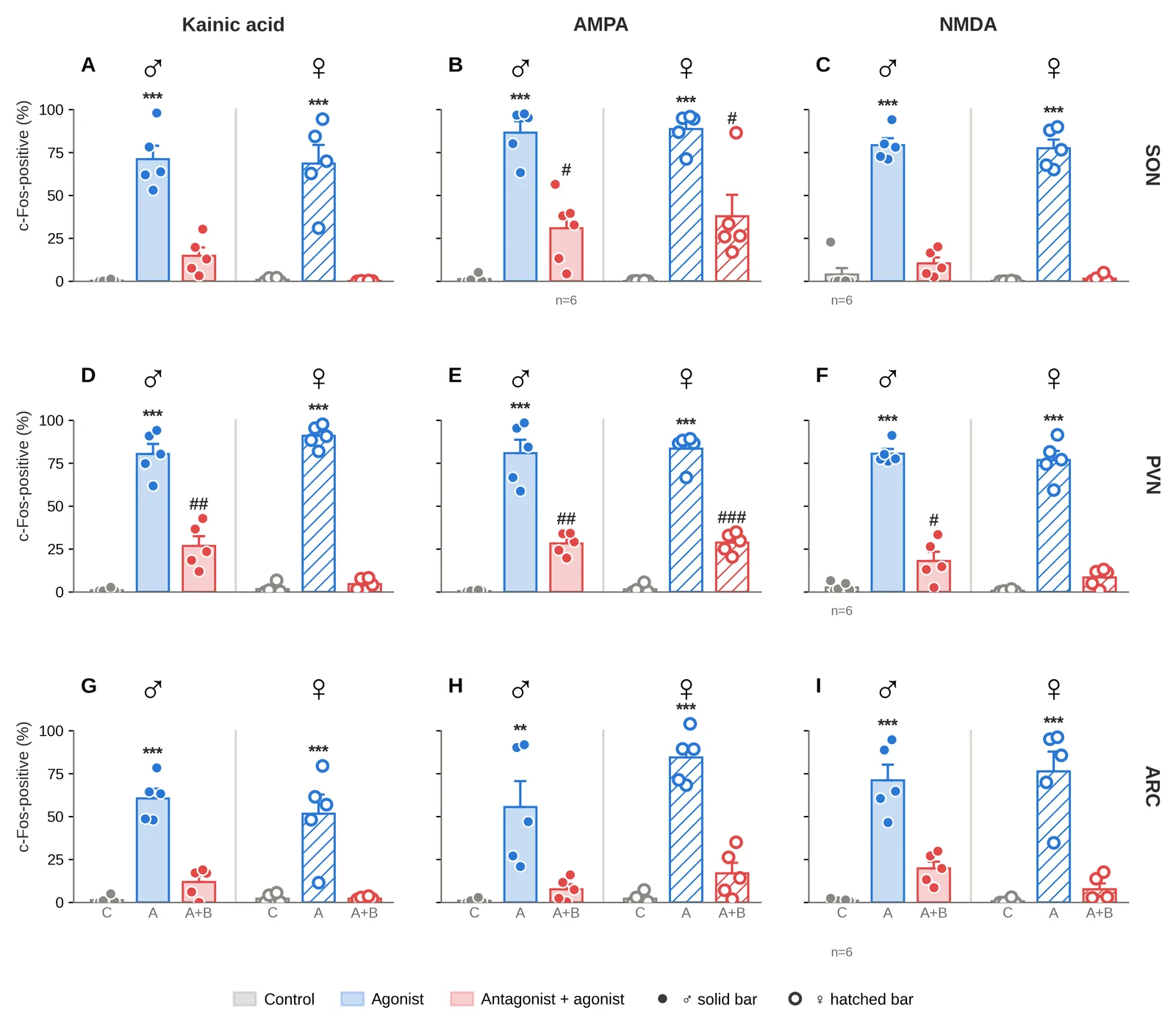
Effects of glutamate receptor agonists and antagonist pretreatment on RSPO1 neuronal activation. The percentage of RSPO1-immunoreactive neurons displaying nuclear c-Fos labeling is shown for the supraoptic nucleus (SON; (**A**–**C**)), paraventricular nucleus (PVN; (**D**–**F**)), and arcuate nucleus (ARC; (**G**–**I**)). Columns represent kainic acid, AMPA, and NMDA treatment, respectively. Within each panel, male groups appear on the left and female groups on the right. C, saline control (gray); A, agonist alone (blue); A+B, antagonist pretreatment followed by agonist administration (red). CNQX was administered 15 min before kainic acid or AMPA, and MK-801 was administered 15 min before NMDA. Solid bars and filled circles indicate males; hatched bars and open circles indicate females. Bars show means with SEM, and each point represents one animal (*n* = 5 per group unless otherwise indicated). Asterisks indicate comparisons between agonist and control groups; hash symbols indicate comparisons between antagonist-pretreated and control groups. One, two, and three symbols denote # *p* < 0.05, ##,** *p* < 0.01, and ###,*** *p* < 0.001, respectively, using one-way ANOVA followed by Tukey’s multiple-comparison test within each sex and treatment paradigm.

**Figure 3:**
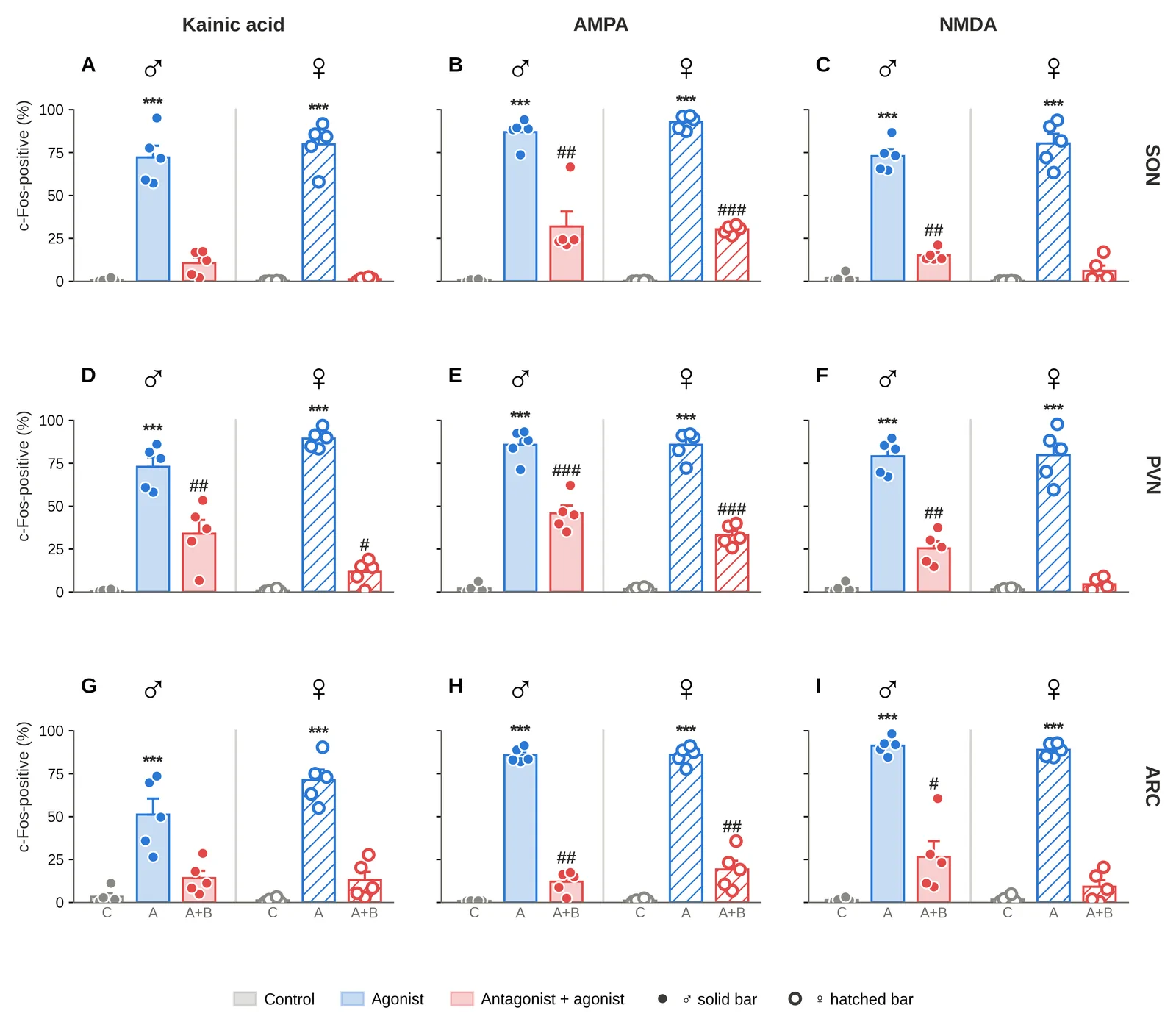
Effects of glutamate receptor agonists and antagonist pretreatment on RSPO3 neuronal activation. The percentage of RSPO3-immunoreactive neurons displaying nuclear c-Fos labeling is shown for the supraoptic nucleus (SON; (**A**–**C**)), paraventricular nucleus (PVN; (**D**–**F**)), and arcuate nucleus (ARC; (**G**–**I**)). Columns represent kainic acid, AMPA, and NMDA treatment, respectively. Within each panel, male groups appear on the left and female groups on the right. C, saline control (gray); A, agonist alone (blue); A+B, antagonist pretreatment followed by agonist administration (red). CNQX was administered 15 min before kainic acid or AMPA, and MK-801 was administered 15 min before NMDA. Solid bars and filled circles indicate males; hatched bars and open circles indicate females. Bars show means with SEM, and each point represents one animal (*n* = 5 per group unless otherwise indicated). Asterisks indicate comparisons between agonist and control groups; hash symbols indicate comparisons between antagonist-pretreated and control groups. One, two, and three symbols denote # *p* < 0.05, ## *p* < 0.01, and ###,*** *p* < 0.001, respectively, using one-way ANOVA followed by Tukey’s multiple-comparison test within each sex and treatment paradigm.

Control activation was low across both RSPO populations, with group means below 4%. Kainic acid, AMPA, and NMDA increased the percentage of RSPO neurons expressing c-Fos in the SON, PVN, and ARC of both sexes. All 36 agonist-versus-control comparisons were significant (Tukey’s HSD, all *p* ≤ 0.003), with agonist group means ranging from approximately 51% to 93% (Figure 2 and Figure 3).

### 2.3. Antagonist Pretreatment Attenuates Activation, Which Remains Above Control in Selected Conditions

CNQX pretreatment reduced kainic acid- and AMPA-induced activation, while MK-801 reduced NMDA-induced activation. All 36 antagonist-plus-agonist versus agonist-alone comparisons were significant (Tukey’s HSD, all *p* ≤ 0.007). Nevertheless, activation remained significantly above control in 17 of 36 antagonist-pretreated conditions. These comprised 10 of 12 AMPA conditions, four of 12 NMDA conditions, and three of 12 kainic acid conditions (Figure 2 and Figure 3).

After AMPA, activation remained above control in both RSPO populations in the SON and PVN of both sexes and in RSPO3 neurons in the ARC. RSPO1 activation in the ARC did not differ significantly from the control in either sex.

After kainic acid, significant elevations above control were confined to the PVN, involving RSPO1 in males and RSPO3 in both sexes. The remaining antagonist-pretreated groups did not differ significantly from their corresponding controls.

After NMDA, activation remained above control in male RSPO3 populations in all three nuclei and in male RSPO1 neurons in the PVN. None of the female antagonist-pretreated groups differed significantly from the control. Overall, above-control activation after antagonist pretreatment was most frequent in the PVN and in males.

### 2.4. Sex Differences in Activation and Antagonist-Mediated Attenuation

**Figure 4:**
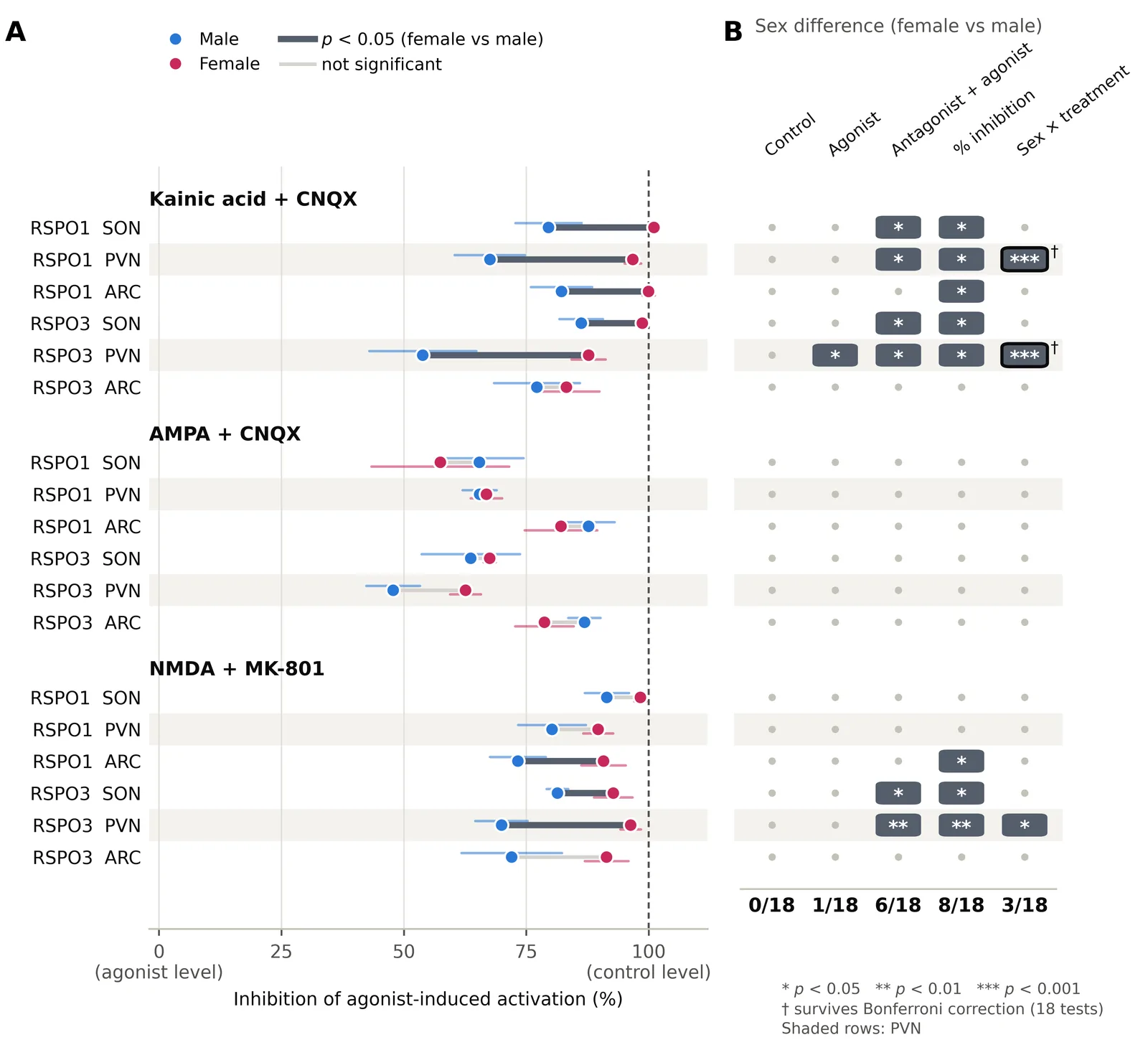
Sex comparisons of RSPO neuronal activation and antagonist-mediated inhibition across 18 peptide × nucleus × agonist conditions. (**A**) Percentage inhibition in males (blue) and females (red), shown as means with SEM of the per-animal values. Percentage inhibition was calculated for each antagonist-pretreated animal as 100 × (Ā − x_i_)/(Ā − C̄), where x_i_ is the percentage of c-Fos-positive RSPO neurons in that animal, and Ā and C̄ are the corresponding sex-specific agonist and control group means [43]. On this normalized scale, 0% corresponds to the agonist group mean and 100% to the control group mean (dashed line). Dark connecting lines indicate *p* < 0.05 for the female–male difference, whereas light lines indicate nonsignificant differences, assessed using Welch’s *t*-test. Rows are grouped by agonist paradigm; shaded rows indicate the PVN. (**B**) Significance map showing between-sex comparisons of control activation, agonist-induced activation, activation after antagonist pretreatment, and percentage inhibition, together with sex × treatment interactions. Between-sex comparisons used Welch’s *t*-test; interactions were assessed using two-way ANOVA. Filled cells indicate *p* < 0.05 and dots indicate nonsignificant comparisons; the numbers below each column give the count of significant comparisons out of 18. One, two, and three asterisks denote unadjusted *p* < 0.05, *p* < 0.01, and *p* < 0.001, respectively. Between-sex comparisons were not adjusted for multiple testing. For the 18 interaction tests, the Bonferroni-adjusted significance threshold was *p* < 0.0028; † indicates interactions that remained significant at this threshold. SON, supraoptic nucleus; PVN, paraventricular nucleus; ARC, arcuate nucleus.

No significant sex differences were detected in the 18 control-group comparisons. Among agonist-treated groups, the only nominal difference was greater kainic acid-induced activation of PVN RSPO3 neurons in females than in males (89.3% versus 72.8%; unadjusted *p* = 0.039). This was an isolated finding among 18 agonist comparisons (Figure 4B).

In antagonist-pretreated groups, six of the 18 comparisons showed significantly lower activation in females. These involved RSPO1 and RSPO3 after CNQX/kainic acid and RSPO3 after MK-801/NMDA in both the SON and PVN. These pairwise sex comparisons were not adjusted for multiple testing (Figure 4B).

Percentage inhibition was also greater in females under selected kainic acid and NMDA conditions, with the largest differences in the PVN (Figure 4A). The largest difference occurred for PVN RSPO3 after CNQX/kainic acid, with inhibition 33.9 percentage points greater in females (95% CI, 4.0–63.7; *p* = 0.033).

**Figure 5:**
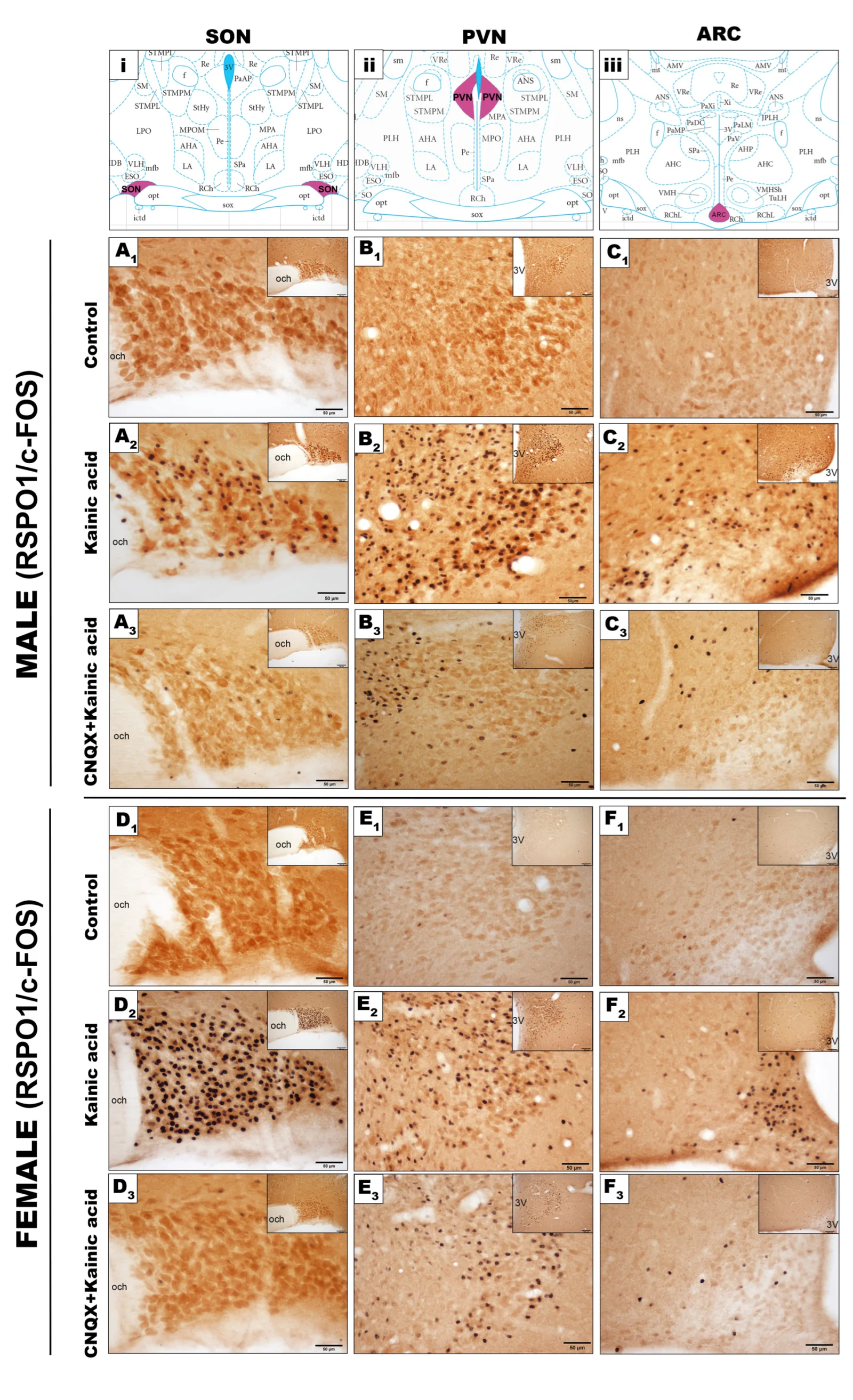
Representative RSPO1/c-Fos labeling following kainic acid administration with or without CNQX pretreatment. Schematic coronal sections indicate the examined supraoptic (SON; i), paraventricular (PVN; ii), and arcuate (ARC; iii) nuclei. Micrographs show male SON (**A_1_**–**A_3_**), PVN (**B_1_**–**B_3_**), and ARC (**C_1_**–**C_3_**), and female SON (**D_1_**–**D_3_**), PVN (**E_1_**–**E_3_**), and ARC (**F_1_**–**F_3_**). Subscripts identify saline control (1), kainic acid alone (2), and CNQX administered 15 min before kainic acid (3). RSPO1 immunoreactivity appears as a brown cytoplasmic DAB reaction product, whereas c-Fos immunoreactivity appears as a blue-black nuclear reaction product following nickel-enhanced DAB staining. Insets show lower-magnification views of the corresponding regions. och, optic chiasm; 3V, third ventricle. Scale bars: 50 µm in the main panels and 100 µm in the insets.

**Figure 6:**
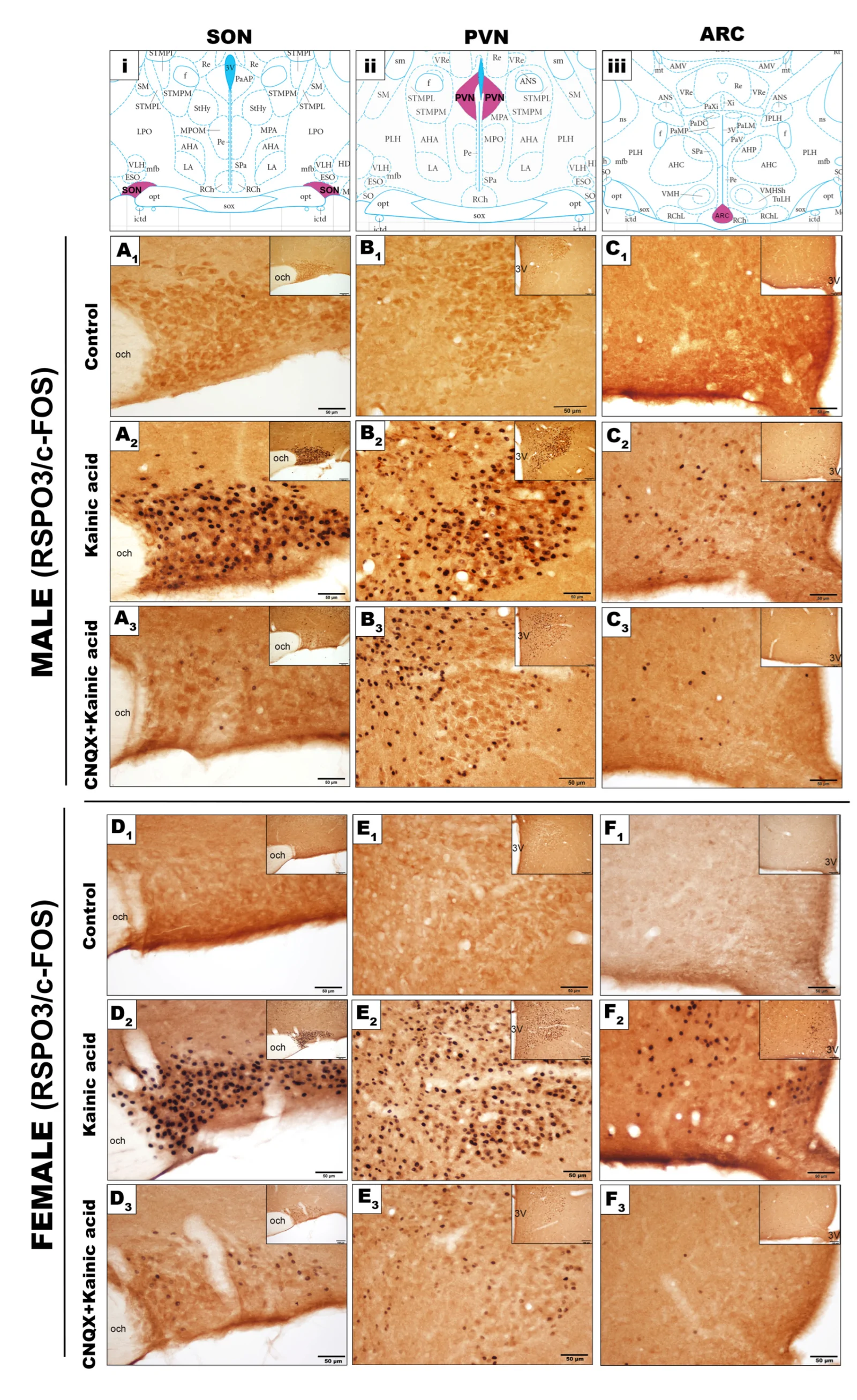
Representative RSPO3/c-Fos labeling following kainic acid administration with or without CNQX pretreatment. Schematic coronal sections indicate the examined supraoptic (SON; i), paraventricular (PVN; ii), and arcuate (ARC; iii) nuclei. Micrographs show male SON (**A_1_**–**A_3_**), PVN (**B_1_**–**B_3_**), and ARC (**C_1_**–**C_3_**), and female SON (**D_1_**–**D_3_**), PVN (**E_1_**–**E_3_**), and ARC (**F_1_**–**F_3_**). Subscripts identify saline control (1), kainic acid alone (2), and CNQX administered 15 min before kainic acid (3). RSPO3 immunoreactivity appears as a brown cytoplasmic DAB reaction product, whereas c-Fos immunoreactivity appears as a blue-black nuclear reaction product following nickel-enhanced DAB staining. Insets show lower-magnification views of the corresponding regions. och, optic chiasm; 3V, third ventricle. Scale bars: 50 µm in the main panels and 100 µm in the insets.

Sex × treatment interactions were detected for PVN RSPO1 and RSPO3 after kainic acid (both *p* = 0.0005) and for PVN RSPO3 after NMDA (*p* = 0.014). Both kainic acid interactions remained significant after Bonferroni correction across 18 tests (α = 0.0028), whereas the NMDA interaction did not. No significant interactions were detected in the SON or ARC (Figure 4B). Representative images from the kainic acid experiments are shown in Figure 5 and Figure 6.

### 2.5. Circulating RSPO Responses to Glutamatergic Treatment

**Figure 7:**
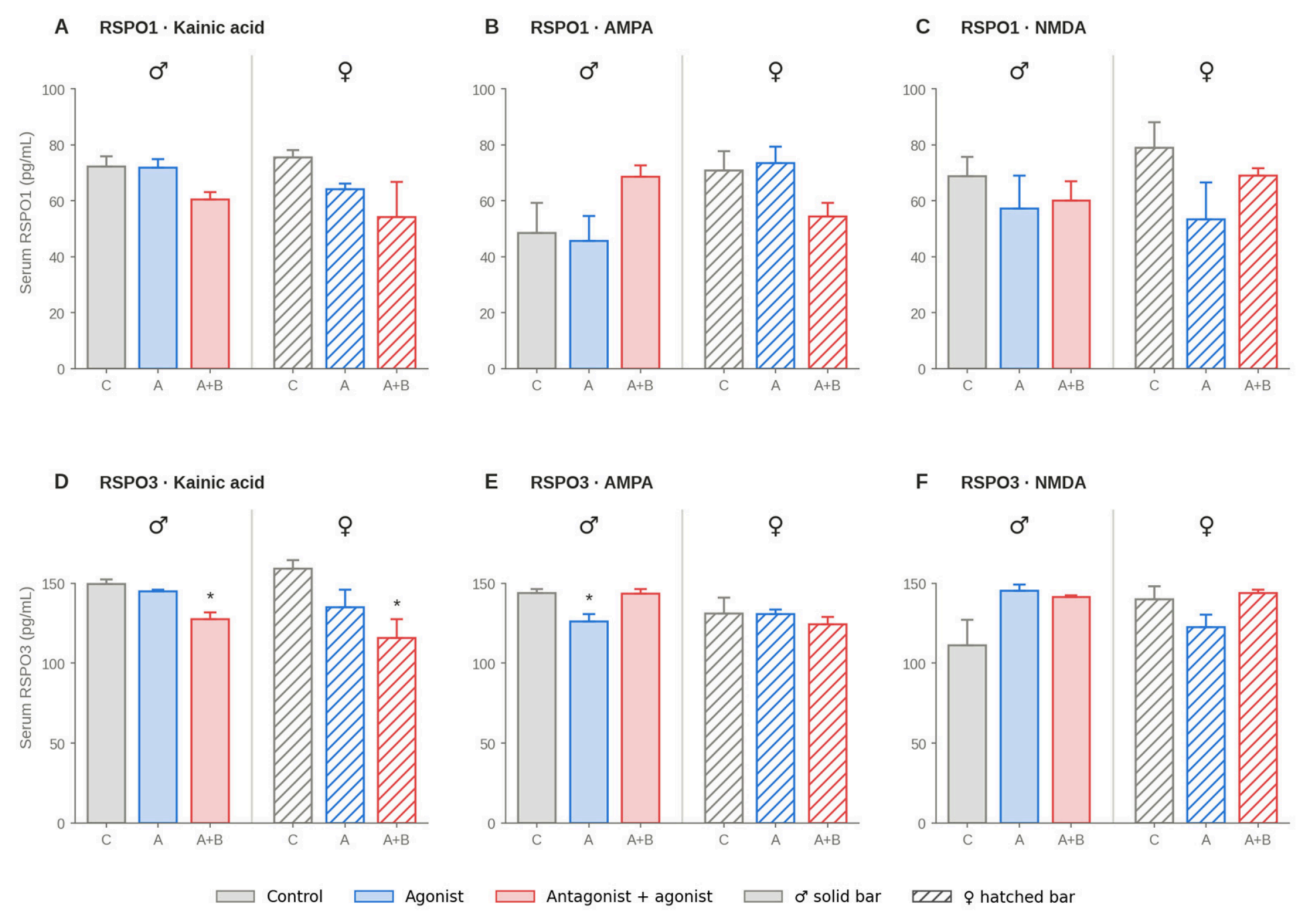
Serum RSPO1 and RSPO3 concentrations following systemic glutamate receptor agonist administration with or without antagonist pretreatment. Serum concentrations measured by ELISA are shown for RSPO1 (**A**–**C**) and RSPO3 (**D**–**F**) following kainic acid, AMPA, and NMDA treatment, respectively. Within each panel, male groups appear on the left and female groups on the right. C, saline control (gray); A, agonist alone (blue); A+B, antagonist pretreatment followed by agonist administration (red). CNQX was administered 15 min before kainic acid or AMPA, and MK-801 was administered 15 min before NMDA. Solid bars indicate males and hatched bars indicate females. Bars show means with SEM. Asterisks indicate *p* < 0.05 versus the corresponding same-sex control, assessed using one-way ANOVA followed by Tukey’s multiple-comparison test. Six male RSPO3 measurements below the lowest assay standard were replaced by their respective group means in the primary analysis. Results of sensitivity analyses excluding these measurements are reported in Section 2.5.

Serum RSPO1 showed no significant pairwise treatment differences in either sex, despite a nominal overall treatment effect in the male kainic acid experiment (one-way ANOVA, *p* = 0.049). For RSPO3, overall treatment effects were detected in the male kainic acid and AMPA experiments and the female kainic acid experiment (Figure 7).

In males, serum RSPO3 was lower after CNQX plus kainic acid than after control treatment (*p* = 0.001) or kainic acid alone (*p* = 0.006). AMPA treatment also produced lower concentrations than control (*p* = 0.014) or CNQX plus AMPA (*p* = 0.016). In females, serum RSPO3 was lower after CNQX plus kainic acid than after control treatment (*p* = 0.030).

Six male RSPO3 measurements fell below the lowest calibration standard and were replaced with the corresponding group mean in the primary analysis. When these measurements were excluded, four of the five significant pairwise differences remained significant. Only the male AMPA versus CNQX-plus-AMPA comparison lost significance (*p* = 0.085).

Changes in serum RSPO concentrations did not consistently parallel neuronal activation. Across individual animals, serum concentrations were not correlated with the mean RSPO/c-Fos activation index (RSPO1, r = −0.14, 95% CI −0.40 to 0.14, n = 53; RSPO3, r = −0.15, 95% CI −0.39 to 0.10, n = 62).

## 3. Discussion

Glutamate receptor stimulation recruited both RSPO populations across the three hypothalamic nuclei, while antagonist pretreatment revealed differences in the extent of inhibition across regions and sexes. Attenuation by CNQX and MK-801 supports the involvement of non-NMDA and NMDA receptor mechanisms in this recruitment. Comparable agonist-induced activation and variable antagonist effects have been reported in hypothalamic nesfatin-1 populations [44], placing the present findings within a broader pattern of glutamatergic regulation of peptidergic neurons. For RSPO populations, the regional and sex comparisons add information about how consistently activation can be reduced under different pharmacological conditions.

Activation remained significantly above control in 17 of 36 antagonist-pretreated conditions, including 10 of 12 AMPA conditions, four of 12 NMDA conditions, and three of 12 kainate conditions. Above-control activation was also most frequent in the PVN. These comparisons distinguish a significant reduction relative to agonist treatment from the degree to which activation approaches control levels. The AMPA findings illustrate this distinction: CNQX attenuated activation, yet labeling frequently remained elevated. Kainate and AMPA were examined using the same CNQX dose in separate treatment groups, making this a useful comparison within a shared antagonist regimen. In hippocampal slices, CNQX has shown similar antagonist potency against AMPA and kainate responses [37]. The present difference therefore characterizes the outcomes of the two stimulation paradigms in the tested hypothalamic populations, rather than establishing a general difference in CNQX efficacy against these receptor classes.

The clearest sex-related pattern appeared after antagonist pretreatment. The only nominal agonist-related difference concerned PVN RSPO3 neurons after kainate, with greater activation in females (89.3% versus 72.8%; unadjusted *p* = 0.039). This isolated finding among 18 comparisons contrasts with the six direct sex differences detected in antagonist-pretreated groups, all showing lower activation in females. These involved RSPO1 and RSPO3 after CNQX/kainate and RSPO3 after MK-801/NMDA in both the SON and PVN. The regional interpretation is strengthened by the two PVN sex-by-treatment interactions in the kainate paradigm, which remained significant after correction across 18 tests. The corrected interaction tests therefore strengthen the PVN findings, while the SON comparisons identify additional populations showing sex-dependent attenuation.

These sex differences matter when antagonist regimens are used to manipulate RSPO-associated circuits: comparable systemic doses can leave different levels of neuronal activation in males and females. The resulting physiological effect could therefore depend partly on the degree of neuronal recruitment achieved in each sex. A focused mechanistic question is whether the differences arise from the glutamatergic inputs reaching RSPO neurons, their receptor properties, or activation through connected populations. Evidence for steroid-sensitive hypothalamic glutamatergic regulation [39,40] provides a rationale for examining these alternatives under controlled hormonal conditions. Such experiments would help distinguish the contribution of hormonal state from other sources of variation in the treatment response.

The location of the effects helps prioritize their functional investigation. The SON findings make neurosecretory responses relevant alongside metabolic outcomes, while the stronger PVN evidence identifies a region in which RSPO regulation could interact with both types of output. The region-dependent hormone-release responses reported after local glutamate receptor manipulation [30] support evaluating the two nuclei separately when linking RSPO activation to endocrine function. For metabolic outcomes, melanocortin-sensitive transmission at PVN MC4R neurons [27,31] provides a candidate circuit in which to examine the consequences of altered RSPO neuronal activity. Testing endocrine and feeding-related responses after selective manipulation of RSPO populations would establish whether the pharmacological differences observed here have functional consequences.

The exploratory HypoMap analysis provides candidate cellular phenotypes for such experiments [45]. In the *Oxt* cluster, approximately 15% of cells had detectable *Rspo1* transcripts and 12% had detectable *Rspo3*, identifying an oxytocin-associated population relevant to the SON/PVN observations. The ligand distributions differed more markedly in *Pomc* clusters, where *Rspo1* was detected in approximately 20–28% of cells and *Rspo3* in approximately 1%. *Agrp* clusters showed corresponding proportions of approximately 8–12% and 4%, while a distinct *Mc4r*/*Fezf1*-defined cluster showed approximately 42% and 7%. These patterns suggest that RSPO1 and RSPO3 do not have interchangeable cellular distributions within the selected neuronal classes. The higher *Rspo1* detection in mouse *Pomc* clusters also contrasts with the sparse coexpression reported in rat POMC neurons by in situ hybridization [18]. Importantly, ligand transcript detection addresses potential RSPO-producing cells, whereas the LGR4/POMC functional literature [18,19] identifies RSPO-responsive circuitry. Considering both observations helps distinguish candidate sources from targets when designing studies of local RSPO signaling. Co-detection of ligand and phenotypic markers provides a starting point for combining RSPO labeling with cell-type-specific recordings, allowing pharmacological responses to be linked to particular neuroendocrine phenotypes.

The serum analyses examined whether neuronal recruitment was accompanied by corresponding changes in circulating RSPOs. Serum RSPO1 showed no significant pairwise treatment differences, and significant RSPO3 changes relative to control were decreases in selected conditions. There was therefore no significant agonist-associated serum increase accompanying the widespread neuronal activation. The selected RSPO3 effects also differed from the broadly distributed RSPO/c-Fos responses, indicating different treatment-response profiles across the two measurements. Under the present conditions, circulating concentrations did not consistently track hypothalamic activation. This result supports assessing the cellular and circulating responses separately when studying systemic glutamatergic challenges. A serum measurement alone would not describe the regional activation pattern identified by RSPO/c-Fos labeling.

This study has several limitations. First, systemic drug administration does not distinguish direct effects on RSPO neurons from responses mediated through other central or peripheral pathways, including upstream neuronal circuits. Although serum RSPO concentrations were measured alongside neuronal activation, these measurements do not resolve this distinction. A single sampling point cannot establish the time course or tissue origin of circulating RSPOs, and the absence of an increase does not establish a central site of drug action. In addition to these limitations concerning the site of action, c-Fos labeling provides evidence of neuronal activation but does not demonstrate RSPO release or subsequent WNT/β-catenin signaling [41,42].

Further characterization is also needed to connect the observed responses to specific neuronal phenotypes. The mouse transcriptomic analysis identifies candidate cell populations for investigation in rats, but it does not directly identify the RSPO populations activated in the present experiments. Moreover, transcript detection depends on sampling and assay sensitivity, and localization of the *Mc4r*/*Fezf1* cluster to the PVN would require spatial evidence. Validation of these cellular identities would therefore help determine which hypothalamic populations account for the pharmacological responses. Finally, establishing the physiological significance of these responses requires functional measurements. Because feeding behavior, reproductive hormones, and reproductive outcomes were not assessed here, their relationship to sex-dependent RSPO neuronal regulation remains to be established.

## 4. Materials and Methods

### 4.1. Animals, Housing, and Ethics

Adult Sprague–Dawley rats aged 61–62 days, comprising females weighing 170–200 g and males weighing 210–240 g, were obtained from the Bursa Uludağ University Laboratory Animal Breeding, Application and Research Unit (DENHAB). Animals were housed four per cage at 20–24 °C with food and water available ad libitum. After acclimatization, animals were individually numbered and randomly assigned to treatment groups within each sex. No separate allocation-concealment procedure was used.

The light–dark cycle was gradually reversed by shifting it by 2 h per day until the dark period extended from 07:00 to 19:00. Animals then adapted to this schedule for approximately 15 days before experimentation.

Females were examined during metestrus to standardize estrous-cycle stage. Vaginal smears were collected using a glass dropper, fixed in methanol for 5 min, stained with hematoxylin and eosin (Bio-Optica Milano S.p.A., Milan, Italy, 05-06002; Bio-Optica Milano S.p.A., Milan, Italy, 05-10007)., and examined by light microscopy as previously described [46]. Only females confirmed to be in metestrus were included.

The planned sample size was based on an a priori power analysis performed in G*Power 3.1.9.7 (one-way ANOVA, fixed effects, nine groups) using an expected effect size of f = 0.45, α = 0.05, and 80% power, which indicated 10 animals per group. The planned design comprised nine treatment groups with five animals of each sex per group, totaling 90 animals.

All procedures were approved by the Bursa Uludağ University Local Animal Experiments Ethics Committee (7 June 2022; approval no. 2022-08/08). Experiments were conducted between 2023 and 2025, and reporting followed ARRIVE 2.0 principles.

### 4.2. Experimental Groups and Drug Administration

Three treatment groups were established for each agonist paradigm: saline control, agonist alone, and antagonist pretreatment followed by agonist administration. Agonist doses were kainic acid (ab120100, Abcam, Cambridge, UK), 2.5 mg/kg in 300 µL; AMPA (ab120005, Abcam, Cambridge, UK), 5 mg/kg in 750 µL; and NMDA (ab120052, Abcam, Cambridge, UK), 100 mg/kg in 2 mL. CNQX (ab120040, Abcam, Cambridge, UK) (2 mg/kg in 300 µL) was administered before kainic acid or AMPA, whereas MK-801 (ab120027, Abcam, Cambridge, UK)(1 mg/kg in 300 µL) was administered before NMDA. All drugs were prepared in physiological saline and administered intraperitoneally at doses used in our previous studies [44,47,48].

Each animal received two injections 15 min apart. Control animals received saline followed by saline, agonist groups received saline followed by the agonist, and antagonist-pretreated groups received the antagonist followed by the agonist. The first injection volume was 300 µL; the second matched the corresponding agonist volume. Injection timing, handling, and total volume were therefore matched within each paradigm.

Injections were administered between 08:00 and 10:00. Animals underwent transcardial perfusion fixation under deep anesthesia with ketamine (Ketalar®, Pfizer, New York, NY, USA) (100 mg/kg, i.p.) and xylazine (Rompun® 2%, Elanco GmbH, Cuxhaven, Germany) (15 mg/kg, i.p.) 90 min after the second injection to assess c-Fos expression [41]. After blood collection, the vasculature was flushed with saline, followed by 300 mL of 4% paraformaldehyde (P6148, Sigma-Aldrich, St. Louis, MO, USA) in 0.13 M phosphate buffer (Merck, Darmstadt, Germany), pH 7.4. Brains were post-fixed overnight at 4 °C. Coronal sections were cut at 40 µm using a vibratome (VT1000 S, Leica Biosystems, Nussloch GmBH, Germany), collected into five series, and stored in cryoprotectant at −20 °C.

### 4.3. Inclusion and Exclusion Criteria

Quantitative analysis required completion of the assigned treatment protocol, adequate perfusion fixation, and intact sections with identifiable target nuclei and evaluable immunostaining. Exclusion criteria included suspected incomplete injection, illness, inadequate perfusion, substantial tissue damage, poor staining, or inability to identify the target nucleus using atlas landmarks.

Section quality was assessed separately for each peptide and nucleus.

### 4.4. Dual Immunoperoxidase Labeling and Controls

RSPO/c-Fos labeling was performed on free-floating sections. Sections were washed in 0.05 M Tris-HCl buffer (Sigma-Aldrich, St. Louis, MO, USA, T1503; Scharlab, S.L., Sentmenat, Barcelona, Spain, AC07742500), pH 7.6, between major steps, except between blocking and primary-antibody incubation. Blocking was performed for 2 h using 10% normal horse serum (Sigma-Aldrich, St. Louis, MO, USA, H1138), which also served as the antibody diluent. For RSPO3/c-Fos labeling, antigen retrieval comprised incubation in 1 mM EDTA (Ethylenediaminetetraacetic acid disodium salt dihydrate), (Sigma-Aldrich, St. Louis, MO, E6635), pH 8.0, at 85 °C for 20 min, followed by 10 min at room temperature.

Sections were first incubated overnight at room temperature with rabbit polyclonal anti-c-Fos (1:20,000; Millipore, ABE457; lot 3585299, Burlington, MA, USA), followed by biotinylated donkey anti-rabbit IgG for 2 h (1:300; Jackson ImmunoResearch, 711-065-152, Jackson ImmunoResearch Laboratories, West Grove, PA, USA). Detection used an avidin–biotin–peroxidase complex for the RSPO1/c-Fos protocol (VECTASTAIN Elite ABC Kit, PK-6100; Vector Laboratories, Newark, CA, USA) or peroxidase-conjugated streptavidin for the RSPO3/c-Fos protocol (1:1000; Jackson ImmunoResearch, 016-030-084, Jackson ImmunoResearch Laboratories, West Grove, PA, USA), each for 1 h. Nuclear c-Fos labeling was developed for 20 min using nickel-enhanced DAB containing 2 g nickel ammonium sulfate (Sigma-Aldrich, St. Louis, MO, USA, A1827), 12.5 mg DAB (Sigma-Aldrich, St. Louis, MO, USA, D5637), and 1.3 µL of 30% H_2_O_2_ (Sigma-Aldrich, St. Louis, MO, USA, H1009) in 50 mL Tris-HCl buffer.

After a second 2 h blocking step, sections were incubated with mouse monoclonal anti-RSPO1 overnight at room temperature (1:3000; clone OTI11A9, IgG2b; OriGene, TA804626, OriGene Technologies, Rockville, MD, USA) or mouse monoclonal anti-RSPO3 for 48 h at room temperature (1:5000; clone OTI3A9, IgG1; OriGene, TA804635, OriGene Technologies, Rockville, MD, USA). Biotinylated donkey anti-mouse IgG was then applied for 2 h (1:300; Jackson ImmunoResearch, 715-065-151, Jackson ImmunoResearch Laboratories, West Grove, PA, USA), followed by the corresponding ABC or streptavidin detection system for 1 h. Cytoplasmic labeling was developed using 12.5 mg DAB and 1.3 µL of the same H_2_O_2_ stock in 25 mL Tris-HCl buffer for 15 min for RSPO1 and 7 min for RSPO3. Sections were mounted, dried, and coverslipped with DPX (Sigma-Aldrich, St. Louis, MO, USA). Procedural negative controls omitted the primary or secondary antibody.

### 4.5. Serum RSPO1 and RSPO3 Measurements

Blood was collected from the right atrium under deep anesthesia before perfusion. Samples were centrifuged at 1000× *g* for 15 min, and serum aliquots were stored at −80 °C. RSPO1 and RSPO3 concentrations were measured using commercial ELISA kits according to the manufacturer’s instructions (YLbiont; RSPO1, YLA1413RA; RSPO3, YLA1480RA, Shanghai YL Biotech Co., Ltd., Shanghai, China).

Manufacturer-reported assay sensitivities were 3.72 pg/mL for RSPO1 and 1.02 pg/mL for RSPO3, with intra-assay and inter-assay coefficients of variation below 8% and 10%, respectively. Samples were assayed in duplicate, with a standard curve included on each plate.

### 4.6. Cell Counting and Quantification

Sections were examined using an Olympus BX-50 (Olympus Corporation, Tokyo, Japan) microscope with a 40× objective, and images were captured with an Olympus DP71 camera using CellSens Imaging Software version 2.3 (Olympus Corporation, Tokyo, Japan). Target nuclei were identified using the rat brain atlas of Paxinos and Watson [49]: SON, bregma −0.48 to −1.44 mm; PVN, −1.32 to −1.92 mm; and ARC, −1.80 to −2.76 mm. Counting used sections from five equidistant rostrocaudal levels, matched in coordinates across animals; all of the approximately 30 serial sections in each vial, taken at 200 µm intervals, were mounted.

Within each selected section, all RSPO-immunoreactive neuronal profiles in the defined nucleus were counted, together with those displaying nuclear c-Fos labeling. Two observers independently performed the counts while blinded to treatment-group identity. Group identities were not disclosed to the observers until microscopic evaluation was completed.

For each animal and nucleus, the activation index was calculated as the number of double-labeled neurons divided by the total number of RSPO-immunoreactive neurons, multiplied by 100. Counts were combined across the analyzed sections before calculating this percentage. The animal was the experimental unit.

### 4.7. Exploratory Analysis of Hypothalamic Transcriptomic Data

Publicly available mouse hypothalamic single-cell transcriptomic data from HypoMap [45] were examined to characterize the cellular distribution of *Rspo1* and *Rspo3* transcripts (CELLxGENE release; 384,925 cells; cell-type labels and the C66 and C185 levels of the HypoMap cluster hierarchy). Cells were scored as transcript-positive when at least one count was detected.

For each annotated neuronal population, the proportion of transcript-positive cells was calculated by dividing the number of cells with detectable *Rspo1* or *Rspo3* by the total number of cells in that population. Separately, the cellular composition of each transcript-positive population was calculated as the proportion assigned to each major cell class. These analyses distinguished expression within a particular cell type from the distribution of expressing cells across cell types. Analyses were performed in Python 3.12.11 using anndata 0.13.4 and pandas 3.0.2, without additional cell filtering.

### 4.8. Statistical Analysis

Data are presented as mean ± SEM. Treatment effects on neuronal activation were assessed using one-way ANOVA followed by Tukey’s honestly significant difference test, separately for each peptide, sex, nucleus, and agonist paradigm. Normality and homogeneity of variance were assessed using the Shapiro–Wilk and Levene tests, respectively.

Sex and treatment effects were assessed using two-way ANOVA with Type III sums of squares for each of the 18 peptide × nucleus × agonist combinations. Bonferroni correction was applied across the 18 sex × treatment interaction tests, giving a significance threshold of 0.05/18. Between-sex comparisons within each treatment group used Welch’s *t*-test and were not adjusted for multiple testing.

Percentage inhibition was calculated for each antagonist-pretreated animal as 100 × (Ā − x_i_)/(Ā − C̄), where x_i_ is the animal’s activation index, and Ā and C̄ are the corresponding agonist and control group means for the same sex, peptide, nucleus, and treatment paradigm [43]. A value of 0% corresponds to the agonist group mean, and 100% to the control group mean. Percentage inhibition was compared between sexes using Welch’s *t*-test.

Serum concentrations were compared using one-way ANOVA followed by Tukey’s test within each sex and agonist paradigm. Six male RSPO3 measurements below the lowest assay standard were replaced by the corresponding group mean in the primary analysis. Sensitivity analyses repeated the comparisons after excluding these measurements.

For each peptide, the association between serum concentration and neuronal activation was assessed using Pearson’s correlation, pairing each animal’s serum measurement with its mean RSPO/c-Fos activation index across the SON, PVN, and ARC.

Except for the corrected interaction tests, statistical significance was defined as *p* < 0.05. Analyses were performed in Python 3.12.11 using SciPy 1.17 and statsmodels 0.15.

## 5. Conclusions

The present findings support NMDA and non-NMDA glutamate receptor involvement in the activation of hypothalamic RSPO1 and RSPO3 neurons. Sex differences were more evident after antagonist pretreatment than after agonist stimulation, with greater attenuation in females in selected SON and PVN conditions and the strongest statistical evidence in the PVN. These neuronal responses were not consistently reflected in circulating RSPO concentrations. The findings therefore identify regional and sex-related differences in the pharmacological regulation of hypothalamic RSPO populations. Defining the neuronal phenotypes involved and determining whether their activation alters RSPO release will help establish the relevance of this regulation to feeding and neuroendocrine function.

## Data Availability

Individual animal-level data and the Python analysis code are available from the corresponding author upon reasonable request. The HypoMap single-cell dataset analyzed in this study is publicly available through CELLxGENE.
